# 

**Published:** 1908-01-25

**Authors:** 


### CONTENTS.

| | PAGE |
|---|---|
| **Social Questions and the Medical Profession** | 435 |
| **Dr. Newsholme, Medical Officer of the Local Government Board** | 436 |
| **Annotations—** | |
| Singing in Relation to Pulmonary Disease—Observations on the Knee-jerk—Professor Koch on Sleeping Sickness | 437 |
| **Medical Opinion and Movement** | 438 |
| **Hospital Clinics—** | |
| Endometritis and the Operation of Curettage. By Thos. G. Stevens, M.D., B.S.Lond., F.R.C.S.Eng., M.R.C.P.Lond. | 439 |
| Asthma in Infants and Young Children. By Edmund Cautley, M.D.(Cantab.), F.R.C.P. Lond. | 442 |
| **Illustrative Cases—** | |
| Acute Nephritis with Extreme Hæmaturia | 445 |
| A Case for Diagnosis | 446 |
| **Points in Surgery—** | |
| Acute Intestinal Obstruction with Abnormal Features | 447 |
| Cysts in the Floor of the Mouth | 448 |
| **Resident Medical Officers' Department—** | |
| Northampton General Hospital | 448 |
| **The General Practitioner's Column—** | |
| The Treatment of Some Common Ailments | 449 |
| **Notes on Current Neurology—** | |
| A Case of Ischæmic Paralysis | 450 |
| **Mental Diseases—** | |
| A Suggested Reform of the Lunacy Laws | 451 |
| **Pathology in General Practice—** | |
| Streptothrix Infections | 452 |
| **The Evolution of Medicine—** | |
| Old Masters of Medicine | 453 |
| **Therapeutics—** | |
| Prescriptions and Dispensing | 454 |
| **Hospital Administration—** | |
| The United Kingdom Hospitals Conference in March | 455 |
| Social and Poor-law Problems | 456 |
| News and Coming Events | 457 |
| "The Tribune" Exhibition of Winter Foods and Drinks | 459 |
| **Nursing Administration—** | |
| The Poor-law Midwifery Schools | 458 |
| **New Appliances and Things Medical—** | |
| The Pulvo | 460 |
| **Editor's Letter-Box—** | |
| Proprietary Preparations | 460 |
| Wanted, Lady Mission Doctors | 460 |

